# Photo-Healable and Stretchable Fibers from Upcycled TPEE and Azopolymers by Harnessing Solid-to-Liquid Photoisomerization

**DOI:** 10.1021/acsami.5c13061

**Published:** 2025-09-01

**Authors:** Yen-Shen Hsu, Tsung-Hung Tsai, Chun-Chi Chang, Tse-Yu Lo, Kai-Chuan Kuo, Yu-Chun Lin, Ji Lin, Kesavan Manibalan, Chia-Wei Chang, Jhih-Hao Ho, Che-Tseng Lin, Jiun-Tai Chen

**Affiliations:** † Department of Applied Chemistry, 34914 National Yang Ming Chiao Tung University, Hsinchu 300093, Taiwan; ‡ Department of Performance Materials Synthesis & Application Division of Polymer Research Material and Chemical Research Laboratories, 63129 Industrial Technology Research Institute, Hsinchu 300044, Taiwan; § Center for Emergent Functional Matter Science, 34914 National Yang Ming Chiao Tung University, Hsinchu 300093, Taiwan

**Keywords:** upcycling, azobenzene, photoinduced, electrospinning, smart-healing fibers

## Abstract

The increasing emphasis on environmental sustainability has driven the development of products derived from recycled plastics; however, their applications remain largely confined to packaging and beverage containers due to high recycling costs and limited economic viability. This study focuses on upcycling plastic waste by depolymerizing and repolymerizing waste polyethylene terephthalate (PET) into thermoplastic polyester elastomers (TPEE). To enhance the functional properties of the resulting material, an azobenzene-containing polymer (PAzo) is incorporated, leveraging its reversible photoinduced solid-to-liquid phase transition under ultraviolet and visible light irradiation. Electrospinning is employed to fabricate one-dimensional photoresponsive fibers composed of a TPEE/PAzo blend. The self-healing capability of the fibers is investigated by evaluating healing efficiency at different PAzo concentrations using tensile testing and analyzing microscopic healing behavior via scanning electron microscopy (SEM). This approach presents a potential strategy for developing high-value, recyclable, and smart-healing fibers.

## Graphical abstract

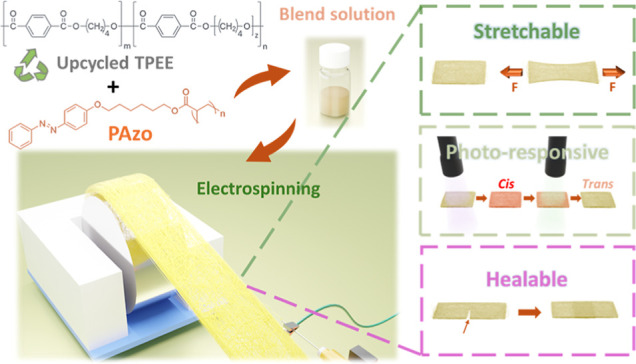

## Introduction

Polymer fabrics have emerged as attractive candidates for next-generation materials due to their breathability, mechanical flexibility, and potential integration into applications such as wearable electronics, soft robotics, and medical textiles.^1–4^ However, the soft and fibrous nature of these materials makes them inherently vulnerable to mechanical damage, which can compromise both functionality and durability.^5,6^ To address this issue, self-healing polymers have been introduced, enabling autonomous or stimuli-triggered repair of physical damage.^7–9^ Among the various external stimuli, light stands out for its spatiotemporal precision, noncontact nature, and wireless controllability, making photoresponsive healing systems a particularly appealing solution.^10–12^

Recent advances in optically healable materials have leveraged dynamic interactions such as noncovalent bonding, metal–ligand coordination, and photoisomerization.^13–15^ Azobenzene, a well-known photoswitchable molecule, undergoes reversible *trans*–*cis* isomerization under UV and visible light, respectively, leading to changes in polarity, geometry, and viscoelastic properties.^16–19^ This photodynamic behavior has been successfully incorporated into polymer systems to regulate adhesion, mechanical performance, and even self-healing.^20–22^ For instance, Liang et al. presented an azobenzene-functionalized polymer as a healable coating that undergoes photoinduced solid-to-liquid transitions, enabling trenchless rehabilitation of damaged pipelines.^23^

In parallel with growing environmental concerns over global plastic waste, both recycling and upcycling approaches have emerged as vital strategies for mitigating the ecological impact of postconsumer plastics.^24^ While recycling typically involves reprocessing materials into products of equal or lower value, upcycling purposes to transform waste materials into products of higher value and functionality, thereby extending their life cycle with added utility.^25^ This distinction has driven increasing interest in the upcycling of polyethylene terephthalate (PET), one of the most used and discarded polymers.^26,27^

In recent years, various upcycling strategies and applications for PET waste have been developed beyond conventional polymer reprocessing. For instance, some studies have demonstrated the electrolytic upcycling of waste PET into value-added chemicals such as acid derivatives and hydrogen fuel, offering a dual benefit of reducing plastic pollution and contributing to sustainable energy solutions.^28,29^ In another example, Gao et al. reported a solvothermal approach for converting waste PET and poly­(vinyl alcohol) (PVA) into PET-based engineering plastics (PEPGs) without the use of catalysts. This method features a relatively simple reaction process and results in PEPGs with excellent mechanical strength and long-term durability, representing a promising route for high-value material recovery.^30^

Thermoplastic polyester elastomers (TPEE), particularly those derived from recycled polyethylene terephthalate (r-PET), offer a promising platform for such efforts due to their elasticity, tensile strength, and melt-processability.^31,32^ For instance, upcycled TPEE has been processed into microfibers via electrospinning techniques, providing flexible, stretchable, and mechanically robust fibrous backbones, which exhibit superior mechanical properties compared to commercial TPEE fabrics and conventional r-PET-based textiles.^33^

However, despite the growing interest in the synthesis and application of TPEE, studies investigating their functional properties and responsiveness to external stimuli remain limited. Notably, very few reports have integrated the concept of upcycling with self-healing functionalities, highlighting a significant gap in the development of sustainable and intelligent polymer systems. To enhance its functional performance and expand its application scope, we present a new class of stretchable, photoresponsive, and self-healable polymer fabrics by blending upcycled TPEE with an azobenzene-containing polymer (PAzo). Serving as a mechanically robust backbone, TPEE provides the necessary strength and elasticity for the fibrous structure, while the incorporation of PAzo introduces light-responsiveness through its phototriggered solid-to-liquid transition (with a glass transition temperature (*T*_g_) of ∼13.7 °C in the *cis* form). This synergistic combination enables the composite fibers to undergo light-induced morphological reconfiguration and efficient damage repair without the need for additional healing agents. Under UV irradiation, localized liquefaction of PAzo facilitates fiber-to-fiber fusion at damaged sites, followed by visible-light-induced resolidification to restore structural integrity. This system not only exemplifies the integration of sustainability and advanced functionality but also offers new opportunities for light-activated self-healing in next-generation functional textile applications.

## Results and Discussion

The preparation of TPEE/PAzo polymer solutions is illustrated in Figure 1a. Owing to its relatively low molecular weight (Figure S3b) and rigid liquid crystalline structure, PAzo alone is difficult to electrospin into continuous fibers. In contrast, TPEE, synthesized via polycondensation of depolymerized recycled PET bottles, exhibits excellent mechanical properties—including high tensile strength and elasticity—but lacks external responsiveness. To overcome these limitations, TPEE and PAzo are blended and dissolved in a trifluoroacetic acid and dichloromethane (TFA/DCM) cosolvent system, followed by electrospinning to fabricate one-dimensional nanofibrous polymer mats. Detailed electrospinning parameters and sample preparation procedures are provided in the Experimental Section. The resulting electrospun TPEE/PAzo blend fibers exhibit both stretchability and photoresponsiveness, making them promising candidates for light-triggered self-healing applications. The healing performance of these fibers was assessed via a lap-shear test under UV and visible light irradiation, as illustrated in Figure 1d.

**Figure 1:**
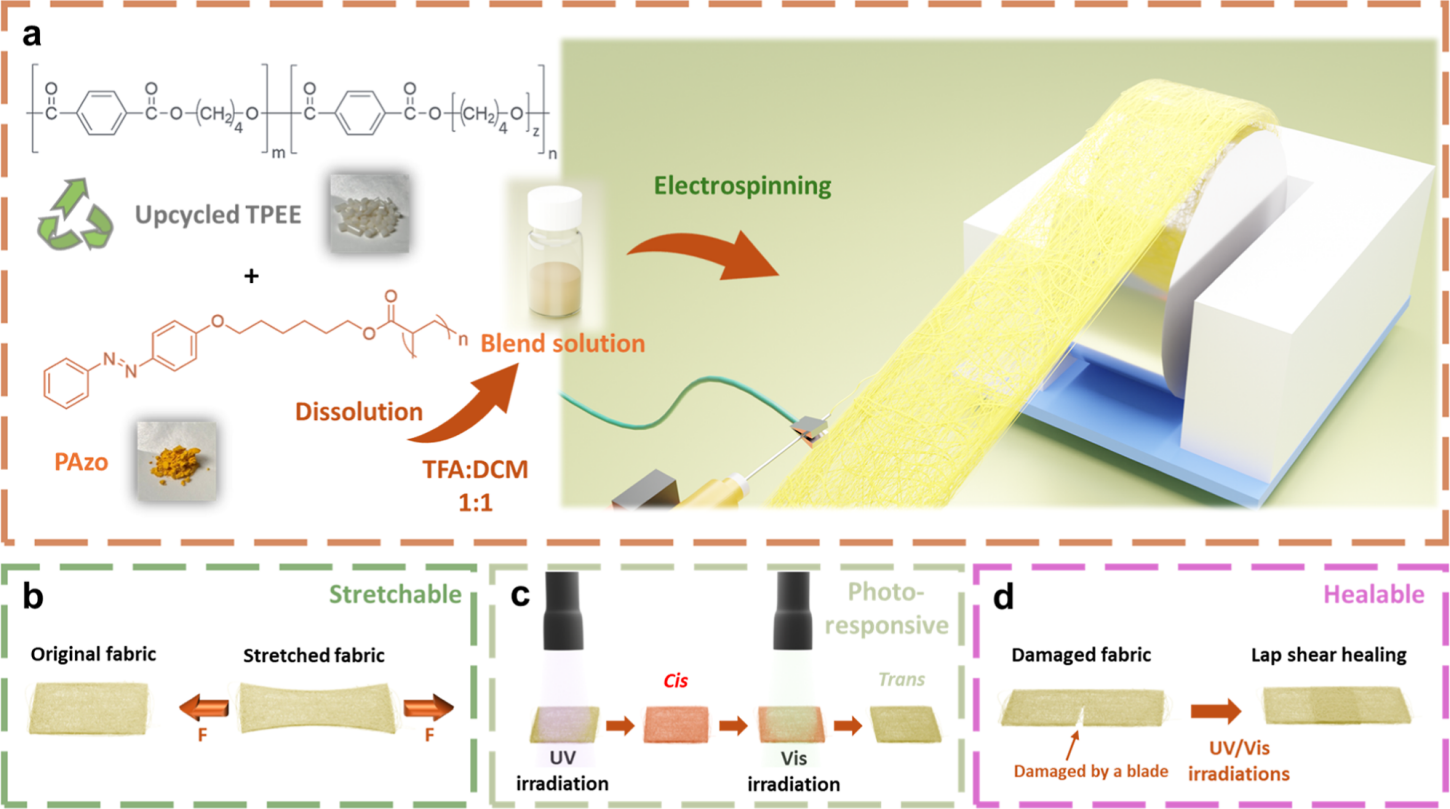
(a) Schematic illustration of the fabrication process of nanofibrous TPEE/PAzo composite fibers via electrospinning of the TPEE/PAzo blend solution. Demonstration of the multifunctional properties of the electrospun fibers, including (b) stretchability, (c) photoresponsiveness, and (d) photoinduced self-healing capability.

The synthetic routes of TPEE are illustrated in Figure 2a. The detailed synthetic procedures and reaction mechanisms are based on our previous study.^33^ Recycled polyethylene terephthalate (PET) was depolymerized with 1,4-butanediol (BDO) in the presence of a titanium-based catalyst under high-temperature nitrogen atmosphere to yield polybutylene terephthalate (PBT) oligomers, which serve as the hard segment of the TPEE. For the soft segment, polytetramethylene ether glycol (PTMEG) was copolymerized with the PBT oligomers via polycondensation, forming long-chain aromatic ester structures. The final product is an upcycled thermoplastic polyester elastomer (TPEE). Additionally, the synthetic route of PAzo is presented in Figure S2a, with structural confirmation by ^1^H NMR shown in Figure S2c.

**Figure 2:**
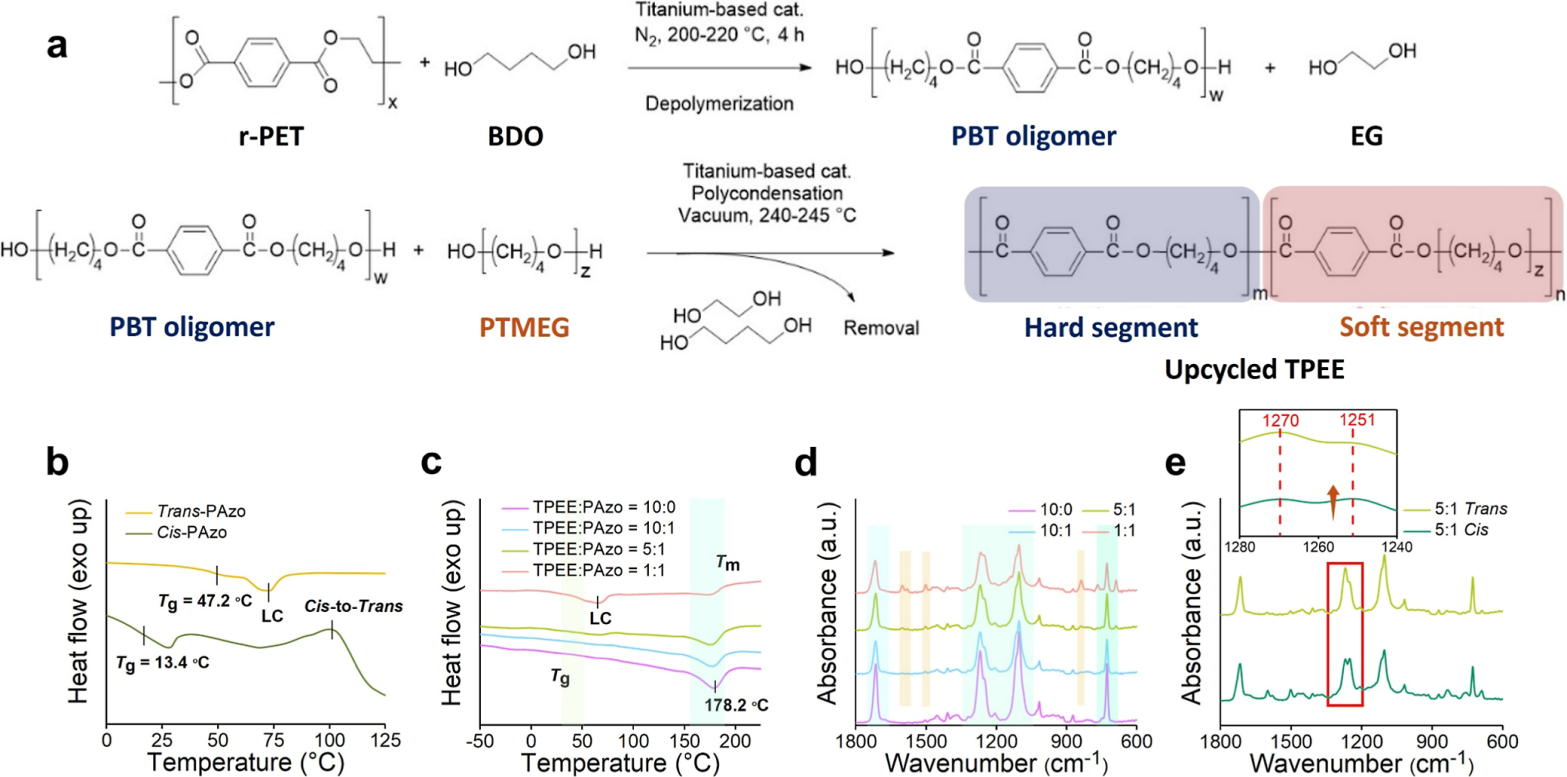
(a) Synthetic routes of TPEE. (b) DSC curves of *trans*-PAzo and *cis*-PAzo isomers. (c) DSC curves of TPEE/PAzo electrospun fibers with different blend ratios: 10:0, 10:1, 5:1, and 1:1. (d) ATR-FTIR spectra of TPEE/PAzo electrospun fibers with varying blend ratios: 10:0, 10:1, 5:1, and 1:1. (e) ATR-FTIR spectra comparing the *trans* and *cis* configurations of the TPEE/PAzo fibers with a 5:1 blend ratio.

The photoinduced reversible solid–liquid phase transition of PAzo arises from the significant difference in *T*_g_ between its *trans* and *cis* isomers. As shown in Figure 2b, the thermal behavior of PAzo is characterized by differential scanning calorimetry (DSC). The *T*_g_ of *trans*-PAzo is observed at 47.2 °C, which is above room temperature, resulting in a solid state. In contrast, the *T*_g_ of *cis*-PAzo is significantly lower, at 13.4 °C, leading to a soft or liquefied state under ambient conditions. Additionally, *cis*-PAzo exhibits thermal relaxation at elevated temperatures (above 100 °C), during which the metastable *cis*-form thermally reverts to the more stable trans-form. Notably, if the *trans*-to-*cis* conversion is incomplete, an endothermic peak corresponding to the liquid crystalline (LC) transition of residual *trans*-PAzo can still be detected at around 75 °C in the DSC thermogram. Figure 2c displays the DSC thermograms of the TPEE/PAzo blend fibers with varying blend ratios. Two thermal transitions near 50 and 75 °C can be clearly distinguished, corresponding to the *T*_g_ of *trans*-PAzo and the liquid crystalline (LC) endothermic peak, respectively. These features are particularly pronounced in the TPEE/PAzo = 1:1 blend, where the PAzo content is highest. An additional melting endotherm is observed at 178.2 °C, which corresponds to the melting temperature (*T*_m_) of TPEE. As the TPEE content increases in the blends, the intensity of the melting peak becomes more prominent, indicating an increased contribution of crystallinity from the TPEE component. Furthermore, the WAXRD results (Figure S4) reveal that the hard segments of TPEE exhibit locally short-range ordered structures, which contribute to the appearance of a stronger endothermic peak.

In addition, we conduct a comparison of the thermal behavior of PAzo and TPEE/PAzo blends before and after UV irradiation, as shown in Figure S5. The phase transition temperatures associated with the PAzo component remain detectable in the blends regardless of irradiation, particularly in the TPEE/PAzo = 1:1 blend fibers, where the PAzo content is highest. This result indicates that the photoresponsive functionality of PAzo is maintained within the TPEE blend, despite the presence of the elastomeric component.

Notably, in Figure S5c, thermal signals corresponding to trans-PAzo can still be observed after UV irradiation. This observation may be attributed to two possible reasons: (1) a small fraction of PAzo may not have undergone complete trans-to-cis photoisomerization; and (2) during the DSC scan from −80 to 250 °C, a portion of the *cis*-PAzo may have thermally relaxed back into the more stable *trans* form. These observations highlight the dynamic nature of the azo-isomerization process and confirm the thermal reversibility of the system.

We also investigate the characteristic peaks of the blended fibers using attenuated total reflectance Fourier-transform infrared (ATR-FTIR) spectroscopy and compare the spectra before and after 20 min of UV irradiation to examine structural changes between the *trans* and *cis* configurations, as shown in Figure 2d,e. Regardless of the TPEE/PAzo blending ratio, characteristic peaks are observed at 1717, 1270, 1105, and 729 cm^–1^, corresponding to C=O stretching, two distinct C–O stretching modes of TPEE, and the out-of-plane bending of aromatic C=C bonds, respectively. In contrast, peaks at 1600, 1502, 1251, and 836 cm^–1^ are attributed to PAzo and show increasing intensity with higher PAzo content. These peaks are assigned to aromatic C=C stretching, N=N stretching, Ar–O–C ether group stretching, and bending vibrations, respectively.

Further comparison of the *trans* and *cis* states in the 5:1 TPEE/PAzo blend (Figure 2e) reveals a noticeable increase in intensity at 1251 cm^–1^ in the *cis* state. This enhancement is attributed to the structural and polarity differences between the *cis* and *trans* isomers, particularly affecting the vibrational behavior of the Ar–O–C ether bond.^34^

The reversibility of the *trans*–*cis* photoisomerization of the PAzo solution is demonstrated in Figure 3a. Initially, before UV irradiation, the PAzo solution is predominantly in the *trans* form, which exhibits a strong π – π* absorption peak at 350 nm. After 20 min of UV irradiation, a weaker n – π* absorption appears at 445 nm, indicating that the solution has been photoinduced into the *cis* form, which absorbs visible light to revert back to the *trans* form. Additionally, Figure S6 shows the changes in absorption intensity over various irradiation times. The results indicate that the *trans*-to-*cis* conversion reaches saturation after approximately 5 min of UV exposure. Upon subsequent visible light irradiation, the intensity of the π – π* absorption band gradually increases as the *cis* form reverts to the *trans* form, with full recovery observed after about 30 min. These findings confirm that the *trans*–*cis* photoisomerization of PAzo is both photoswitchable and reversible.

**Figure 3:**
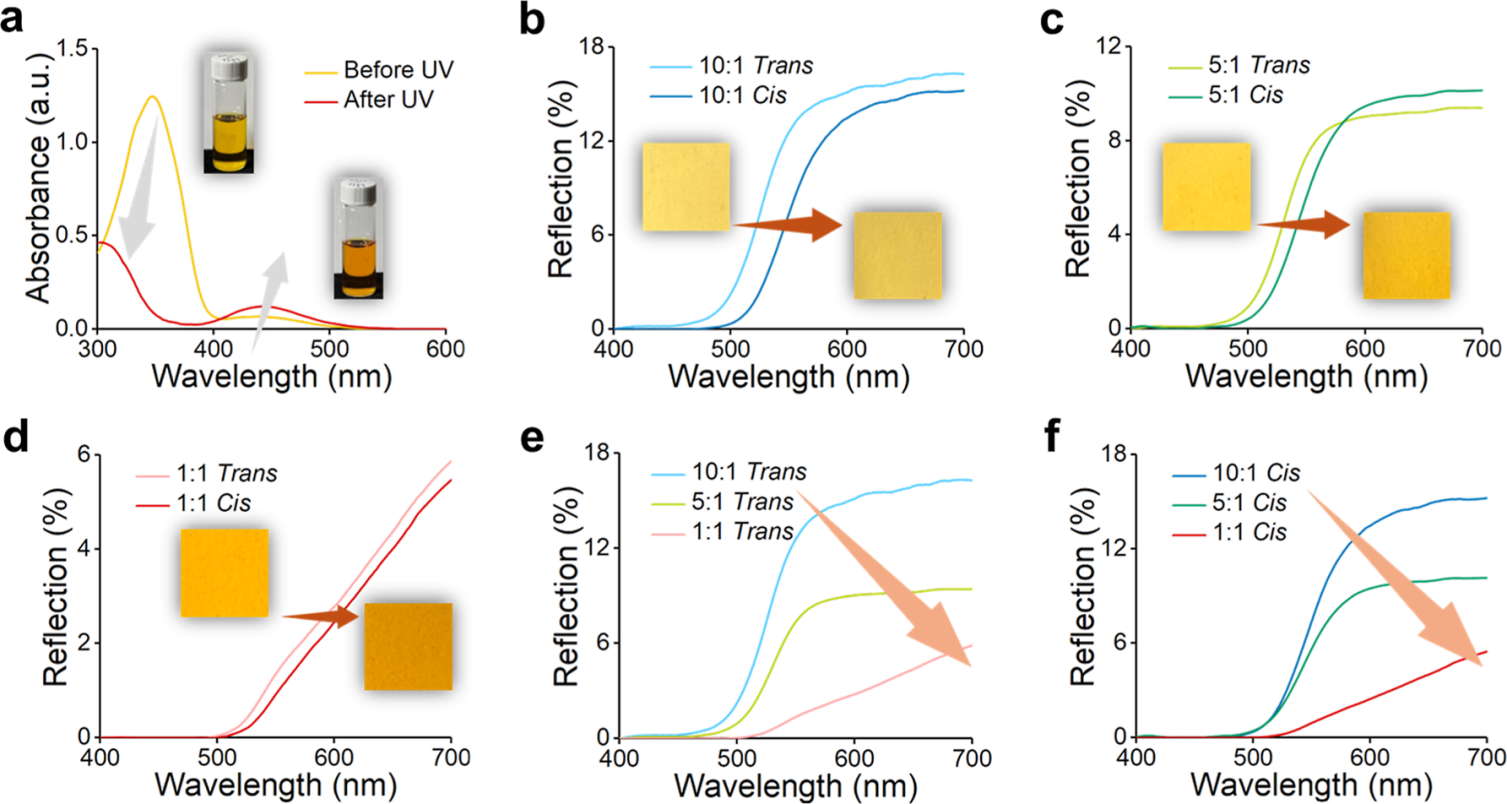
(a) UV–vis absorption spectra of the PAzo solution in THF before and after UV irradiations. (b–d) UV–vis reflection spectra of the TPEE/PAzo blend fibers with different blend ratios before and after UV irradiations: (b) 10:1, (c) 5:1, and (d) 1:1. (e,f) Comparison of TPEE/PAzo blend fibers with different blend ratios before and after UV irradiations: (e) *trans* state (before UV) and (f) *cis* state (after UV).

Because the thickness of the electrospun fiber mats produced in each batch is approximately 50–100 μm, UV–Vis absorption measurements of the blended fibers may be affected by variations in thickness, which could influence the absorption gradient. Therefore, we analyze the reflectance spectra of the fibers instead, as shown in Figures 3b–d and S7. For the three blend ratios (TPEE/PAzo = 10:1, 5:1, and 1:1), but not for the pure TPEE fibers, a redshift in the reflectance spectra is observed after 20 min of UV irradiation, indicating the photoisomerization of PAzo from the *trans* to the *cis* form. This spectral shift corresponds to a visible color change from orange-yellow to reddish-orange. Furthermore, as the PAzo content increases, the redshift becomes more pronounced, with the *cis*-state spectra shifting to longer wavelengths. This result suggests a deeper color tone and a decrease in overall reflectance percentage, as illustrated in Figure 3e,f.

Figure 4 shows the morphologies of the TPEE/PAzo blend fibers with various blend ratios, examined via scanning electron microscopy (SEM). The upcycled TPEE granules and PAzo are dissolved in a mixture of trifluoroacetic acid and dichloromethane (TFA/DCM, v/v = 1:1) at a total polymer concentration of 20 wt %. TFA provides good solubility for TPEE and ensures the formation of a homogeneous and mobile polymer solution. DCM is added as a cosolvent to promote rapid solvent evaporation during electrospinning and to suppress potential side reactions due to the acidic nature of TFA.

**Figure 4:**
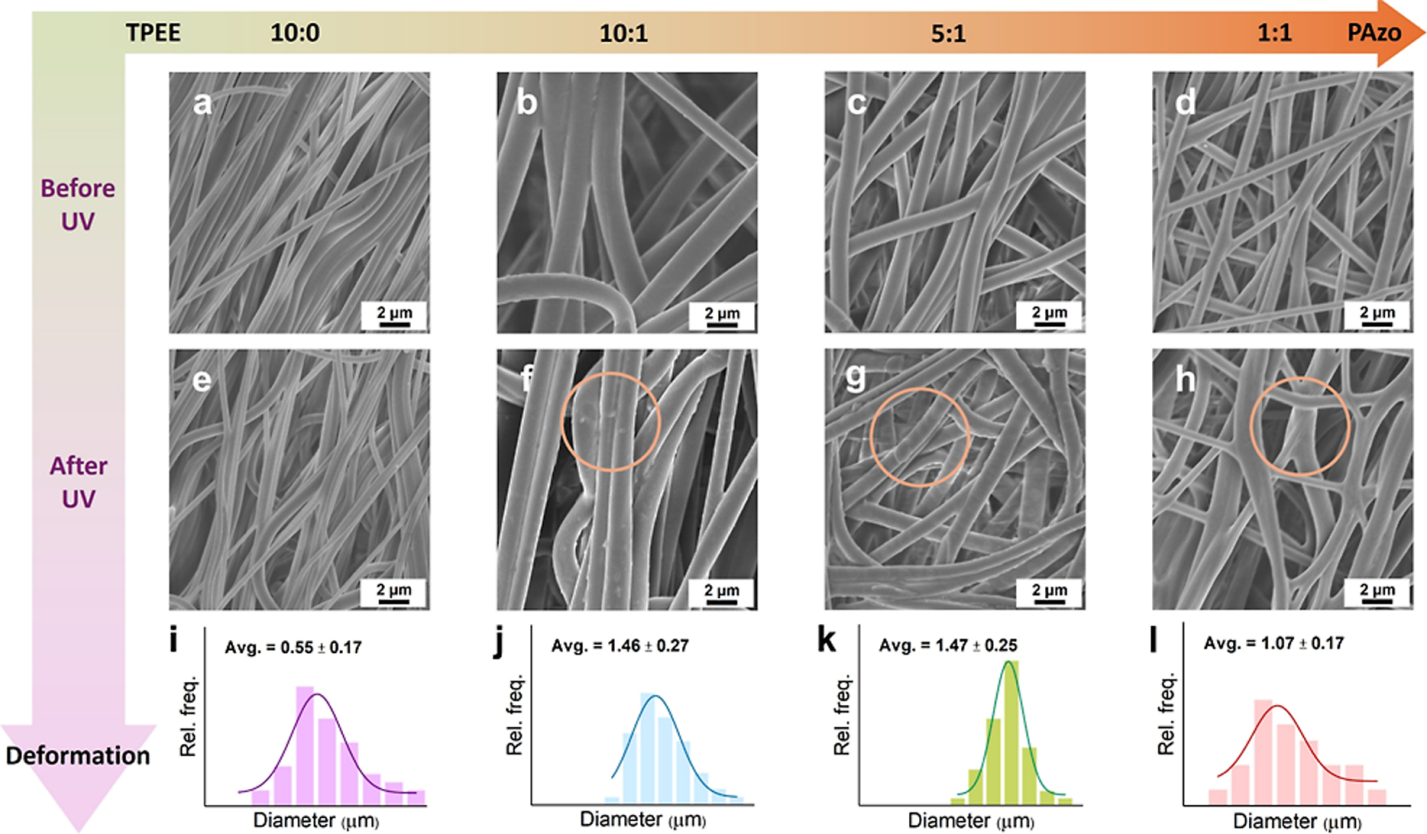
SEM images of electrospun TPEE/PAzo blend fibers with various blend ratios before and after UV irradiations. Blend ratios of TPEE/PAzo are (a,e) 10:0; (b,f) 10:1; (c,g) 5:1; and (d,h) 1:1. Images (a–d) show the fiber morphologies before UV irradiation, while (e–h) show the corresponding fibers after 20 min of UV exposure. Diameter distributions of the electrospun fibers for each blend ratio are displayed in the top-right corner of panels (a–d).

The diameter distribution of the electrospun fibers is analyzed using ImageJ software, revealing that the diameters of individual TPEE/PAzo blend fibers range from approximately 0.55–1.5 μm (Figure 4a–d). When comparing the morphologies of the fibers before and after UV irradiation, pure TPEE fibers (TPEE/PAzo = 10:0) show no morphological changes due to the absence of any photoresponsive groups (Figure 4a,e). In contrast, the blend fibers with TPEE/PAzo ratios of 10:1, 5:1, and 1:1 display noticeable surface deformations, such as bumps and wrinkles, after 20 min of UV irradiation (Figure 4b–d,f–h). These changes are attributed to the *trans*-to-*cis* isomerization of PAzo, which transitions into a soft, liquefied state with increased molecular mobility and viscoelasticity, thus causing subtle topographical modifications on the fiber surface.

To evaluate the mechanical properties of the TPEE/PAzo blend fibers prior to photohealing experiments, three different blending ratios (TPEE/PAzo = 10:1, 5:1, and 1:1) are cut into strips (3 cm × 1 cm) and subjected to uniaxial tensile testing at a stretching rate of 3 mm/min. The stress–strain behavior of the fibers is examined under three states: before UV irradiation (*trans* form), after UV irradiation (*cis* form), and after subsequent visible light irradiation (*trans*–*cis*–*trans* cycle), as shown in Figure 5a–d.

**Figure 5:**
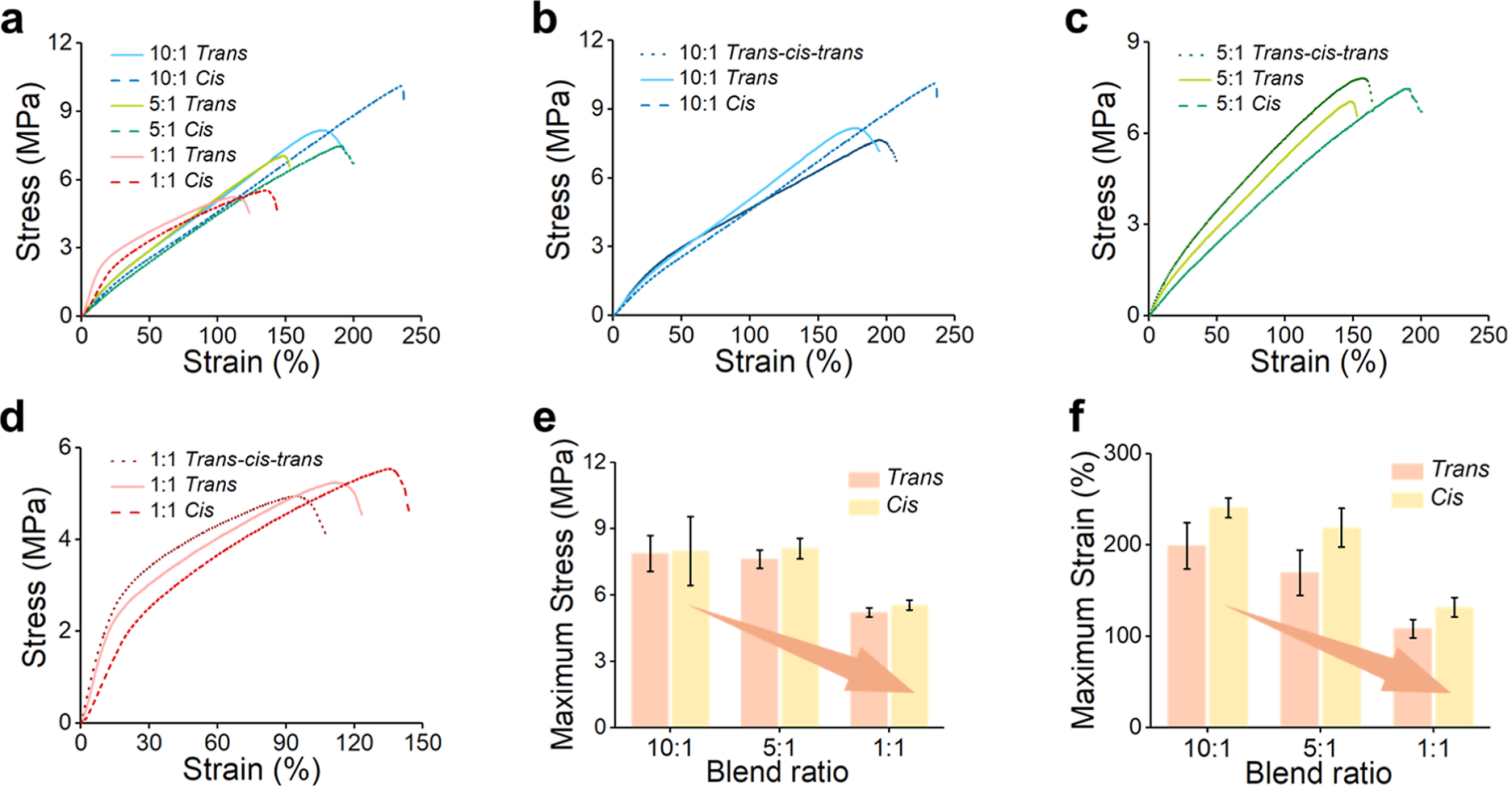
(a) Stress–strain curves comparing *trans* and *cis* states of the TPEE/PAzo blend fibers with three different blend ratios. (b–d) Stress–strain comparisons of *trans*, *cis*, and *trans–cis–trans* states for TPEE/PAzo blends with ratios of (b) 10:1, (c) 5:1, and (d) 1:1. (e,f) Bar charts showing the (e) maximum stress and (f) maximum strain values of the TPEE/PAzo blend fibers before and after UV irradiations for different blend ratios.

Figure 5a overlays the *trans* and *cis* stress–strain curves of the three blends. The 10:1 blend demonstrates the highest tensile strength and elongation at break (∼7.9 MPa, ∼200%), attributed to the elastic nature of TPEE, consistent with our previous studies. As the PAzo content increases (Figure 5b,c), both tensile strength and elongation gradually decrease. Notably, the 1:1 blend exhibits a two-stage modulus (strain = ∼0–15 and ∼15–110%), suggesting mechanical fatigue or yielding behavior after a certain strain threshold.

Upon UV irradiation, converting PAzo to its *cis* form, all blend fibers exhibit increased strain and improved ductility compared to their trans counterparts (Figure 5e,f). This result is likely due to the enhanced viscoelasticity of *cis*-PAzo, which facilitates fiber elongation under stress. This trend is also supported by rheological data (Figures S8 and S9), where the tan θ value increases with higher PAzo content, and further increases upon isomerization to the *cis* form. This outcome indicates a decrease in torsional resistance during oscillatory shear at 10 rad/s at 20 °C under 20 mm parallel plate geometry.

Overall, as the PAzo content increases, both tensile strength and strain decrease significantly (from ∼7.9 to ∼5.2 MPa and from ∼200 to ∼110%, respectively). Importantly, the comparison between the trans and trans–cis–trans conditions shows no significant differences in mechanical performance for the 10:1 and 5:1 blend fibers. However, in the 1:1 blend fibers, the mechanical properties are not fully restored after the trans–cis–trans irradiation cycle. This incomplete recovery is attributed to the partial fluidization of PAzo domains under UV light, which enhances local chain mobility and induces redistribution of the PAzo regions, as evidenced by AFM images in Figure S10. Upon subsequent visible-light irradiation, the cis-PAzo reverts to its more rigid trans form, leading to recovery of rigidity. However, due to the prior redistribution of PAzo domains during the UV-induced cis state, the resolidified trans-PAzo may no longer retain its original morphology or alignment. This structural rearrangement ultimately results in diminished mechanical performance compared to the pristine (unirradiated) fiber, with the effect being particularly pronounced in fibers with the highest PAzo content.

Furthermore, the long-term mechanical stability of the fiber is evaluated by Tensile Strength/Strain Retention (TSR), calculated using the following equation^35,36^

1 $$TSR\left(\%\right)=\frac{residual\;tensile\;strength/strain\;afteraging\;\left(X_{aged}\right)}{tensile\;strength/strain\;of\;unaged\;control\;\left(X_{unaged}\right)}\times 100\%$$

where *X*_aged_ = maximum stress or maximum strain of the fiber after 180 days of storage; *X*_unaged_ = maximum stress or maximum strain of the unaged fiber. The TSR results are summarized in Table 1.

**Table 1:** TSR Comparison for the TPEE/PAzo Blend Fibers with Different Blend Ratios after 180 days of Ambient Storage

	maximum stress (%)		maximum strain (%)	
TPEE/PAzo blend ratio	trans	cis	trans	cis
10:1	53.7 ± 8.6	59.7 ± 11.7	78.6 ± 10.4	80.4 ± 4.4
5:1	66.8 ± 7.1	71.5 ± 11.1	74.6 ± 11.8	70.8 ± 7.8
1:1	65.1 ± 11.0	66.2 ± 6.2	70.6 ± 10.8	63.9 ± 5.6

As shown in Tables S1 and S2, the stress–strain profiles of the fibers after 180 days—both in the *trans* and *cis* forms—exhibit similar trends to those of the pristine blends. In both cases, the maximum stress and strain decrease with increasing PAzo content. However, the absolute values in the aged samples are reduced. For instance, in the 10:1 TPEE/PAzo blend, the maximum stress decrease from ∼7.9 to ∼4.2 MPa, while the maximum strain drops from ∼200 to ∼156%. This degradation is attributed to the slightly hydrophilic nature of TPEE, which undergoes mild hydrolytic degradation^26^ upon prolonged exposure to moisture in the air, resulting in reduced mechanical strength. In terms of TSR comparison, the aged fibers retain approximately 50–70% of their original maximum stress and 60–80% of their maximum strain, indicating a slight decrease in Young’s modulus. This observation demonstrates that long-term air exposure can influence fiber performance—a challenge that must be addressed in future development. Notably, because the TSR for strain shows better retention than that for stress, the healing efficiency discussed in Figure 6 is evaluated based on the ratio of maximum strain.

**Figure 6:**
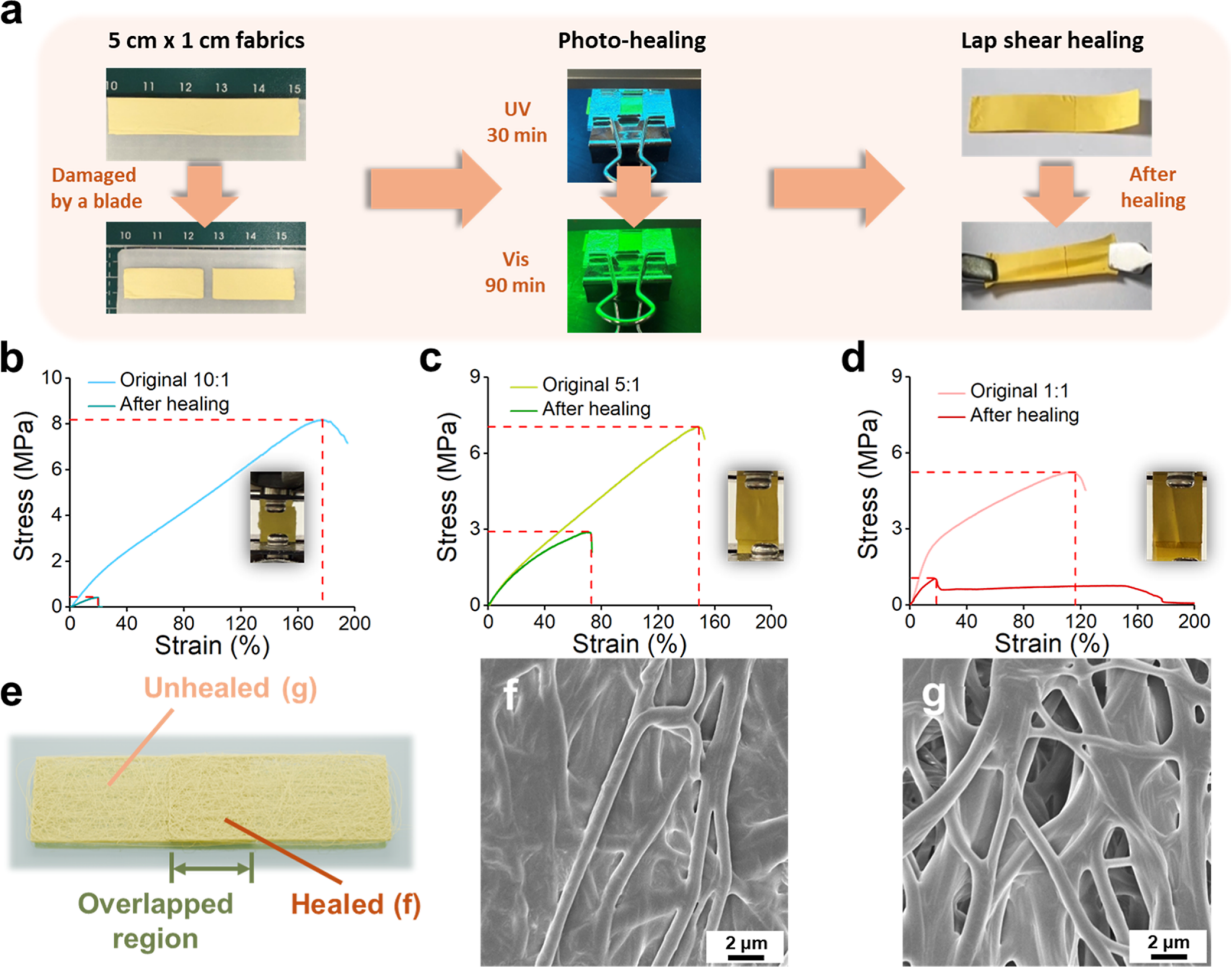
(a) Photographic sequence of the lap shear healing process: a 5 cm × 1 cm TPEE/PAzo blend fiber strip is cut at the center and realigned with a 1 cm × 1 cm overlapping region, fixed using PFA film and aluminum-covered glass slides with a 1 cm light exposure window. (b–d) Tensile stress–strain curves comparing the original (undamaged) and healed fibers for different TPEE/PAzo blend ratios: (b) 10:1, (c) 5:1, and (d) 1:1. (e) Schematic illustration of selective photohealed and unhealed regions during lap shear healing. (f,g) SEM images of the fiber interface after the healing process: (f) photohealed region showing fused morphology and (g) unhealed region without interfacial fusion.

Based on the observed surface morphology changes and tensile test evaluations, we demonstrate the self-healing capability of the photoresponsive smart fibers through a lap shear test, aiming to assess the healing performance of the blend fibers under light stimulation. Figure 6a illustrates the experimental setup: a 5 cm × 1 cm strip of blend fiber is cut at the center using a thin stainless steel blade. Two damaged strips are then overlapped at the cut region by 1 cm × 1 cm and sandwiched between glass slides, secured with clips. A layer of perfluoroalkoxy alkane (PFA) film is used to prevent electrostatic adhesion of the fiber to the glass surface, while aluminum foil partially covers the slides to block light exposure except for a 1 cm-wide window over the overlapping region. The sample is irradiated with UV light for 30 min to induce the *trans*-to-*cis* isomerization of PAzo, enabling it to soften and flow into the damaged area. This process is followed by visible light irradiation for 90 min to revert PAzo to the solid *trans* state, thereby completing the photoinduced healing process, as shown in Video S1.

Figure 6b–d compares the mechanical properties of the TPEE/PAzo blend fibers before damage (undamaged) and after photoinduced lap shear healing. The healing efficiency is calculated by dividing the maximum strain of the healed sample by that of the undamaged fiber (Table S3). For the TPEE/PAzo blend ratio of 10:1, the limited amount of PAzo provides insufficient photoresponsive healing reagent to bridge the damaged interface under stress, resulting in a low healing efficiency of only 11.8%. In contrast, the 1:1 blend exhibits a noticeable drop in stress at the transition between the two-stage Young’s modulus regime during the tensile test. Although the fiber does not completely fail until a strain of ∼150%, this early stress drop indicates that the fiber could no longer withstand increasing applied stress. This observation indirectly confirms the viscoelastic nature of *cis*-PAzo under low stress, making it a viable light-responsive healing reagent.

For the 5:1 blend, a balanced composition provides enough upcycled TPEE to serve as a robust backbone supporting mechanical stress (∼3 MPa), while incorporating a sufficient amount of PAzo to allow efficient healing. As a result, the highest healing efficiency of 50.7% is achieved. These findings highlight that the healing behavior is dependent on an optimal balance between the structural support from TPEE and the dynamic mobility of PAzo, rather than simply increasing the PAzo content.

Interestingly, Table S3 also reveals that the photohealing efficiency of fibers aged under ambient conditions for 180 days is higher than that of their unaged counterparts. This trend may result from slight degradation of the TPEE backbone over time, while the PAzo moiety, which exhibits good hydrophobicity (Figure S11), is less prone to hydrolysis. Notably, the aged fibers show a substantial decrease in their maximum strain capacity, retaining only 60–80% of their original values, as also reported in Table 1. Consequently, the calculated healing efficiency, which is based on the recovery of mechanical performance, appears artificially elevated in aged fibers. However, this enhancement does not represent a proportional improvement in absolute mechanical properties. In addition, the TPEE/PAzo blend with a 1:1 ratio exhibits a larger error margin, indicating greater variability and reduced stability in healing performance. The result could be related to increased PAzo content, which may lead to more pronounced rearrangement and phase separation during the photoinduced liquefaction and healing process. This trend is also reflected in the mechanical data shown in Figures 6d and S12c, further supporting this interpretation.

Figure 6e presents a schematic of the light-exposed and unexposed regions during the lap shear healing process. SEM observations of the light-exposed (healed) area (Figure 6f) show a fused morphology, where *cis*-PAzo—exhibiting high viscoelasticity and mobility at room temperature—flows into and fills the interfiber gaps. Subsequent visible light irradiation reconverts cis-PAzo to its trans form, solidifying the healed interface. In contrast, the unexposed area (Figure 6g) shows no such interfiber fusion, with PAzo failing to migrate and fill the voids, confirming that healing occurs only in the selectively irradiated region.

To further demonstrate the practical applicability of the TPEE/PAzo blend fibers, we conduct an additional experiment to evaluate their breathability and water resistance, both of which are essential properties for wearable and medical textile applications.^37^ As shown in Video S2 and Figure S13, the fabric is placed over the neck of a filtering flask, and water is poured onto its surface. High-pressure N_2_ gas is then introduced through the side port of the flask. The formation of air bubbles in the overlying water confirms that air can pass through the fabric, indicating its breathability. Furthermore, no water leakage is observed either immediately after water is added or after the setup is maintained at room temperature for 18 h. Although a reduction in water height is noted, this is attributed to evaporation from the top surface, as no water droplets are detected in the lower flask. These results confirm that the TPEE/PAzo fabrics possess both breathability and water resistance, underscoring their potential as candidate materials for wearable devices and medical textiles.

In addition, we also assess the mechanical durability of the polymer fabrics before and after UV irradiation by performing cyclic tensile tests, in which samples are stretched to a maximum strain of 50% over 20 cycles. As shown in Figure S14c, the stress response at 50% strain in both the trans and cis states exhibited only a slight decrease over the course of 5–20 cycles. The durability test suggests that the TPEE/PAzo blend fibers maintain mechanical stability under repeated deformation, further reinforcing the potential of the fibers for practical textile applications.

Building on these demonstrations of functionality and durability, our study can be further contextualized within the broader landscape of photoinduced self-healing material. Table 2 summarizes representative studies over the past decade on photoinduced self-healing materials, highlighting their healing performance and associated limitations. Compared to these prior works, the system developed in this study features a relatively simple material composition and an efficient light-driven healing protocol. Specifically, localized liquefaction of the damaged region is triggered by UV irradiation, followed by resolidification upon visible light exposure, with the entire healing process completed within approximately 2 h of total irradiation time.

**Table 2:** Healing Performance and Limitations of Related Photo-Healing Studies in the Past Decade

reference	materials	healing treatment	evaluations of photohealing performance	limitations
this work	TPEE/PAzo blend fibers	UV/vis-induced reversible solid–liquid transition	fibers stretched over 50% of original length during lap shear healing; healed regions selectively activated under SEM	the fibers do not undergo complete mechanical recovery
Lee et al.^38^	poly(2-cinnamoyloxyethyl) methacrylate (PCEMA) film	UV cross-linking at 365 nm and de-cross-linking at 254 nm under continuous heating at 80 °C	the scratches are healed under OM	long photoirradiation (24 h) is required, and the healing wavelength range is challenging to achieve in practical applications
Zhu et al.^39^	microsphere contains xanthate-diamino monomer, EXEP initiator, and benzoquinone inhibitor	blue light irradiation to activate the photopolymerization reaction	the microspheres collapse into a healable coating under SEM	prolonged complete collapsed time (18–24 h); the healing process is irreversible
Chen et al.^20^	PS/PAzo based blend fibers	UV/vis-induced reversible solid–liquid transition	fiber entanglement observed in the T-shaped healing test under SEM	poor mechanical properties of PS; the healing process is lengthy

Moreover, the healing performance is comprehensively evaluated through both morphological and mechanical analyses. Scanning electron microscopy (SEM) reveals clear morphological changes at the damaged region after selective light exposure, confirming that localized repair has occurred. In addition, tensile testing is employed to quantitatively assess the healing efficiency, providing a robust interpretation of both the photohealing capability and the mechanical recovery of the blend fibers. These findings not only validate the photoresponsive healing behavior of the upcycled material but also shed light on potential limitations and challenges in achieving complete structural restoration, offering deeper insight into the design of photohealing polymer systems.

## Conclusions

In this study, we successfully develop novel photohealable and stretchable fabrics by blending upcycled TPEE, synthesized via polycondensation from postconsumer plastic waste, with PAzo synthesized via free radical polymerization (FRP). This carefully designed combination enables the TPEE/PAzo blend fibers to exhibit excellent stretchability, while the PAzo component undergoes reversible *trans*–*cis* photoisomerization under ultraviolet (UV) light. This photoinduced transformation triggers a solid-to-liquid phase transition, facilitated by the notably low *T*_g_ of PAzo (∼13.7 °C), allowing it to function as a healing agent within the fiber network. Subsequent exposure to visible light reverses the isomerization, restoring the solid state and stabilizing the healed structure. The core innovation of this work lies in the photoresponsive self-healing mechanism, which leverages the mobility of liquefied PAzo domains within the TPEE/PAzo fiber matrix. Tensile lap-shear tests conducted on fibers with varying TPEE/PAzo ratios demonstrate that the 5:1 composition achieves the highest healing efficiency, with strain recovery reaching 50.7%. This two-step, light-triggered healing strategy effectively repairs damaged fiber while preserving mechanical integrity. The synergistic integration of plastic waste upcycling with reversible photoresponsive phase transitions presents a promising pathway toward the development of sustainable, functional textiles with extended lifespans.

## Experimental Section

### Materials

Recycled polyethylene terephthalate (r-PET) waste films (impurity grade <100 ppm), trimmed from biaxially oriented polyethylene terephthalate (BOPET), were utilized as the r-PET source. 1,4-Butanediol (BDO) and trifluoroacetic acid (TFA) were obtained from Alfa Aesar, and polytetramethylene ether glycol (PTMEG, *M*_w_ = 1000 g/mol) was purchased from Sigma-Aldrich. Upcycled thermoplastic polyester elastomers (TPEE) with a weight-average molecular weight of 62 kg/mol was synthesized by condensation polymerization according to our previous study,^33^ as presented in Figure 2a. Aniline (99%), anisole (99%), and acryloyl chloride (96%) were purchased from Alfa Aesar. Hydrochloric acid (>37%), phenol (>99%), sodium nitrite (99%), potassium carbonate (99.995%), and potassium iodide (>99.5%) were obtained from Sigma-Aldrich. 2,2′-Azobis­(2-methylpropionitrile) (AIBN, 99%) was supplied by Aencore. 6-Chloro-1-hexanol was acquired from Nova Materials. Ethanol, isopropyl alcohol (IPA, 99.5%), acetone (99%), and anhydrous dichloromethane (DCM, 99.8%) were purchased from Echo Chemical. Sodium hydroxide (NaOH) and tetrahydrofuran (THF) were obtained from Tedia. Azobenzene-containing polymer (PAzo) with a weight-average molecular weight of 7 kg/mol was synthesized via free radical polymerization (FRP), following a previously reported method,^20^ as illustrated in Figure S2a.

### Fabrication of TPEE/PAzo Blend Fibers with the Electrospinning Method

First, upcycled TPEE granules and PAzo were weighed at weight ratios of 10:0, 10:1, 5:1, and 1:1, and placed into glass sample vials. The polymers were then dissolved in a trifluoroacetic acid/dichloromethane (TFA/DCM) mixture (v/v = 1:1) at a total concentration of 20 wt %. The vials were sealed with parafilm to minimize solvent evaporation. The mixtures were stirred at 180 rpm for 24 h at room temperature. Subsequently, 3.5 mL of each prepared solution was loaded into a 5 mL plastic syringe fitted with a horizontally oriented capillary nozzle (inner diameter = 0.41 mm). The syringe was mounted on a syringe pump (KD Scientific) with a set flow rate of 1.0 mL/h. Electrospinning was carried out using a rotating drum collector (diameter = 10 cm, rotation speed = 1000 rpm) covered with parchment papers. The distance between the nozzle tip and collector was fixed at 10 cm. A high-voltage power supply (SMICO) provided a 16 kV bias to initiate the electrospinning process. After approximately 4 h of fiber collection, the resulting electrospun mats were dried under ambient conditions for 24 h.

### Fabrication of the TPEE and r-PET Films with the Spin-Coating Method

The film preparation process is as follows: TPEE and r-PET were each dissolved in TFA to prepare 10 wt % solutions. The solutions were stirred at room temperature for 12 h to ensure complete dissolution. Subsequently, the solutions (0.3 mL) were drop-cast onto 2 × 2 cm glass substrates and spin-coated at 500 rpm for 1 min, followed by 3000 rpm for another 1 min. The coated films were then placed under vacuum for 6 h to remove residual TFA.

### Photoinduced *Trans*–*Cis* Transition of TPEE/PAzo Blend Fibers

A strip of TPEE/PAzo blend fibers was cut into dimensions of 1 cm × 3 cm using a stainless steel blade. The sample was then irradiated with ultraviolet (UV) light (∼365 nm, 700 mW cm^–2^) for 20 min to induce the isomerization of the PAzo component into its *cis*-form. To revert the *cis*-form back to the *trans*-form and achieve solidification, the fibers were subsequently exposed to visible light (∼520 nm, 370 mW cm^–2^) for another 20 min.

### Mechanical and Self-Healing Tests of TPEE/PAzo Blend Fibers

The mechanical properties of the electrospun fabrics were evaluated using a tensile testing machine (Shimadzu, EZ). Samples were cut into 1 cm × 3 cm strips and subjected to the photoinduced isomerization process described in the previous section. After photoactivation, the fibers were mounted onto 3 cm × 8 cm support plates with a central testing area of 1 cm × 2 cm for tensile measurement. For the healing tests, 1 cm × 5 cm fiber mats were cut at the center, and the damaged sections were overlapped by 1 cm × 1 cm. The overlapped region was sandwiched between two glass slides lined with perfluoroalkoxy alkane (PFA) thin film (thickness = 0.025 mm) and secured with clips. The samples were exposed to UV light (∼365 nm) for 30 min to induce the *cis*-form transition of PAzo, causing local liquefaction and fusion at the overlapped interface. Subsequently, visible light (∼520 nm) was applied for 90 min to revert the PAzo to its *trans*-form, promoting solidification of the fused region. Tensile testing was then performed at a constant strain rate of 3 mm/min. The strain at failure (%) was compared between pristine (undamaged) and healed samples to assess the self-healing efficiency of the TPEE/PAzo blend fibers.

### Analysis and Characterization

The morphologies of the electrospun fibers were examined using a scanning electron microscope (SEM, JEOL JSM-7401F) at the micrometer scale. Prior to imaging, the samples were sputter-coated with approximately 4 nm of platinum using a JEOL JFC-1600 sputter coater (20 mA, 50 s) to enhance surface conductivity. SEM measurements were conducted at an accelerating voltage of 5 kV to visualize the surface morphologies of the fibers. Additionally, surface topography were analyzed using an atomic force microscope (AFM, Innova) operated in soft tapping mode. UV–visible (UV–vis) reflection spectra of the fabrics were recorded in the range of 400–700 nm using a Hitachi U-4100 spectrometer. UV–vis absorption spectra of PAzo in solution (0.25 mM in tetrahydrofuran, THF) were measured from 300 to 600 nm using a U-2900 spectrometer.^1^H nuclear magnetic resonance (^1^H NMR) spectra were acquired using a JEOL ECZ500R/S1 spectrometer. Attenuated total reflectance Fourier-transform infrared (ATR-FTIR) spectroscopy (Bruker ALPHA II) was performed to analyze the chemical structure of the electrospun fabrics. Differential scanning calorimetry (DSC, TA Q200) was performed over a temperature range of −80 to 250 °C at heating and cooling rates of 10 °C/min using ∼1 mg samples. Gel permeation chromatography (GPC) was used to determine the apparent weight-average molecular weights of the polymers. GPC measurements were performed using a JASCO RI-4030 refractive index detector coupled with an AS-4050 autosampler. For TPEE, poly­(methyl methacrylate) (PMMA) was used as the calibration standard and trifluoroethanol (TFE) was used as the mobile phase; for PAzo, polystyrene (PS) was used as the calibration standard and tetrahydrofuran (THF) was used as the mobile phase. The flow rate for both GPC measurements was set at 0.1 mL/min. Wide-angle X-ray diffraction (WAXRD, Bruker D8 Discover X-ray diffraction system) was employed to analyze the TPEE and r-PET films. The measurements were carried out over a 2θ range of 5–90°, with a step size of 0.03°.

## Supplementary Material







## Notes

^1^H NMR spectra of TPEE, PAzo, and PAzo monomer; UV–vis reflection spectra of TPEE fibers before and after UV irradiation; GPC chromatograms of TPEE and PAzo polymers; WAXRD analysis of TPEE and r-PET films; DSC curves of TPEE/PAzo blend fibers after UV exposure; UV–vis absorption spectra of PAzo solution under UV and visible light exposure over varying time intervals; UV–vis reflection spectra of pure TPEE fibers before and after UV irradiations; rheology data of TPEE/PAzo blend fibers displaying storage moduli (*G*′) and loss moduli (*G*″) in different times; AFM images of TPEE/PAzo blend fibers before and after UV irradiations; cyclic tensile test data of fabrics before and after UV irradiations (PDF)
Video S1 is the healing demonstration of TPEE/PAzo blend fibers (MP4)
Video S2 is the breathability and water resistance test for TPEE/PAzo blend fibers (MP4)
