# Supplementary material for: Photo-Healable and Stretchable Fibers from Upcycled TPEE and Azopolymers by Harnessing Solid-to-Liquid Photoisomerization
Source: ACS Appl Mater Interfaces. 2025 Sep 1;17(36):51256–66. doi: 10.1021/acsami.5c13061 (PMC12442005; doi:10.1021/acsami.5c13061)
Supplement: Supplementary file 1 [file am5c13061_si_001.pdf]

Supporting Information

Photo-Healable and Stretchable Fibers from Upcycled TPEE and Azopolymers by Harnessing Solid-to-Liquid Photoisomerization

Yen-Shen Hsu,[1] Tsung-Hung Tsai,[1] Chun-Chi Chang,[1] Tse-Yu Lo,[1] Kai-Chuan Kuo,[2] Yu-Chun Lin,[1] Ji Lin,[1] Kesavan Manibalan,[1] Chia-Wei Chang,[1] Jhih-Hao Ho,[1] Che-Tseng Lin,[2] and Jiun-Tai Chen[1,3]*

[1]Department of Applied Chemistry, National Yang Ming Chiao Tung University, 300093 Hsinchu, Taiwan

[2]Department of Performance Materials Synthesis & Application Division of Polymer Research Material and Chemical Research Laboratories, Industrial Technology Research Institute, Hsinchu 300044, Taiwan

[3]Center for Emergent Functional Matter Science, National Yang Ming Chiao Tung University, 300093 Hsinchu, Taiwan

*To whom correspondence should be addressed. E-mail: jtchen@nycu.edu.tw.

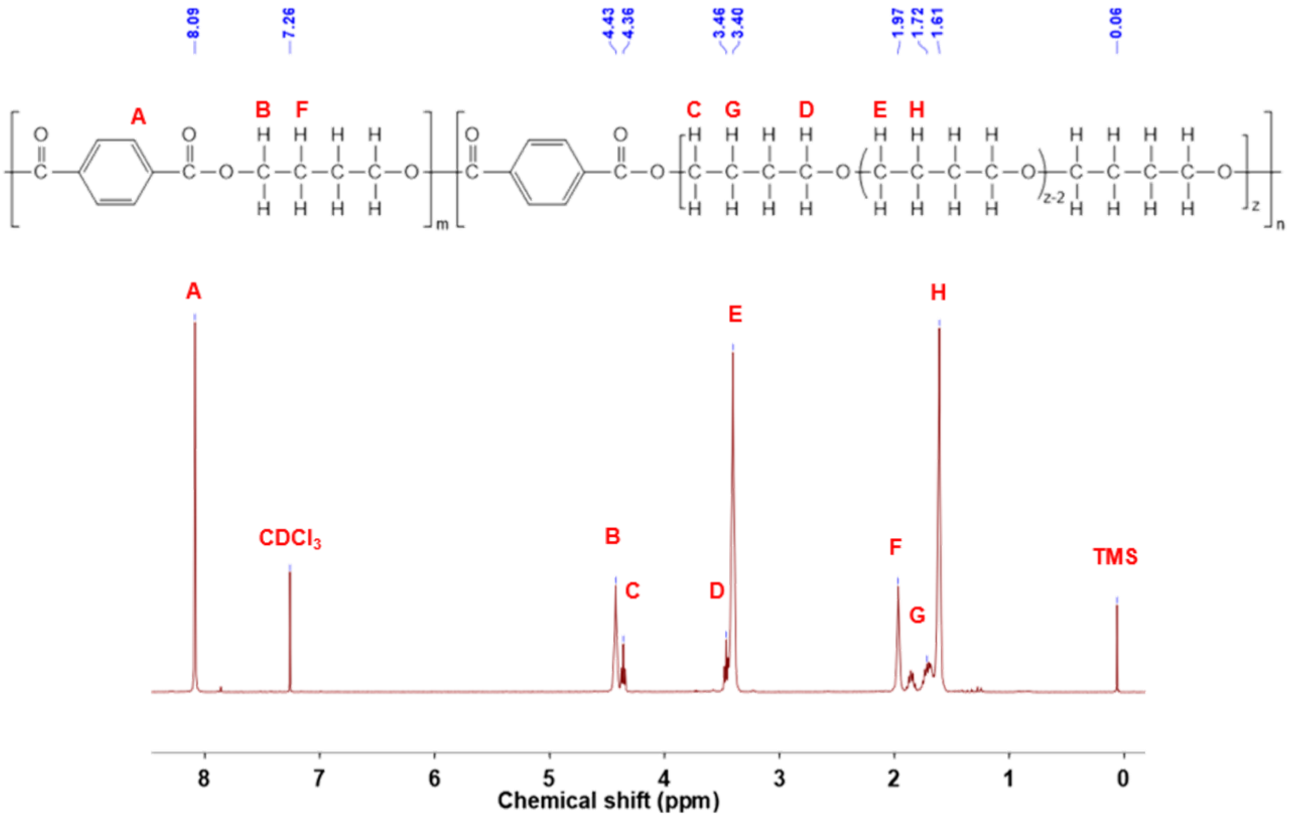

Figure S1. ^{1}H nuclear magnetic resonance (^{1}H NMR) spectrum of TPEE.

The detailed synthetic procedures and reaction mechanisms of PAzo are based on our previous study.[1] The synthesis of PAzo began with a diazotization reaction of aniline, sodium nitrite, and phenol to yield an azo intermediate (Azo1). Azo1 then underwent an SN2 substitution with 6-chloro-1-hexanol to form Azo2. Subsequently, Azo2 was esterified with acryloyl chloride under stirring at room temperature for 20 h. The crude product was extracted and purified using column chromatography with a mixed eluent of ethyl acetate and hexane (1:9 v/v), affording the monomer Azo3. Finally, the PAzo polymer was obtained through free radical polymerization (FRP) of Azo3. The corresponding structural characterizations, including ^{1}H NMR and GPC analyses, are provided in Figure S2b-c and Figure S3b.

(a) Synthesis of PAzo

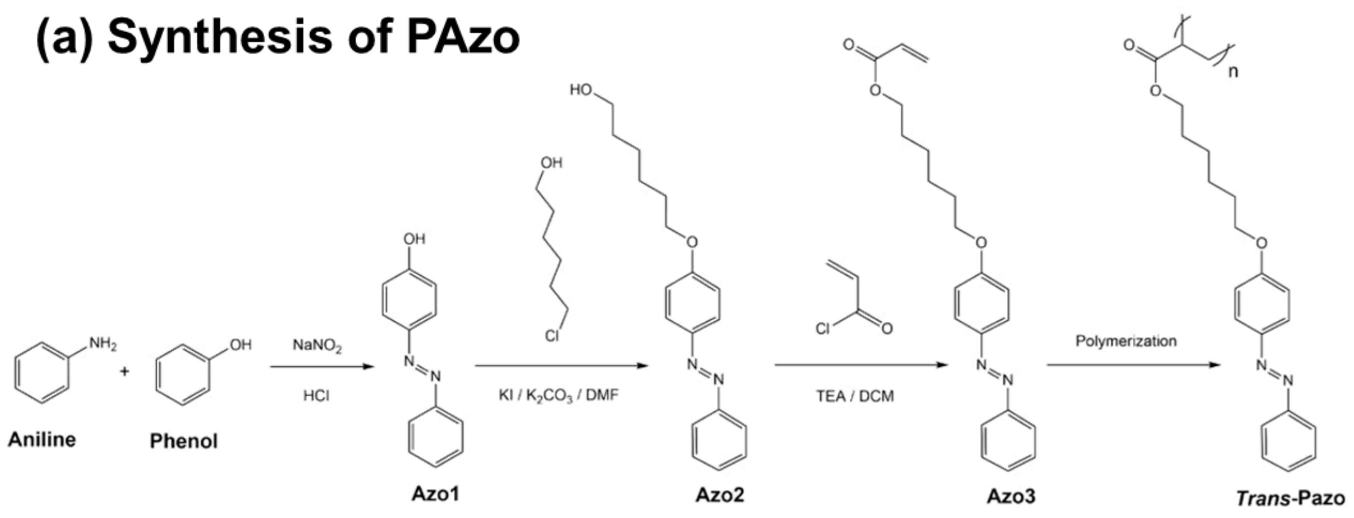

(b) Azo3

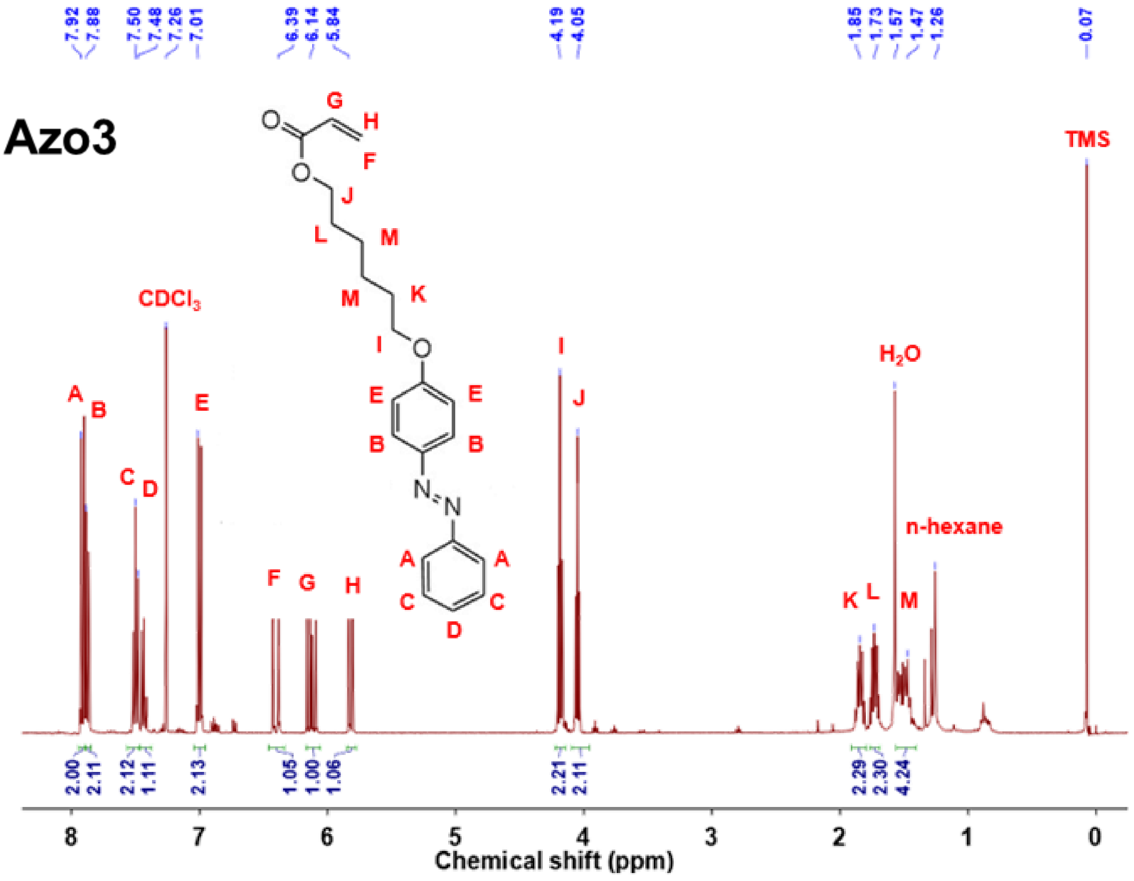

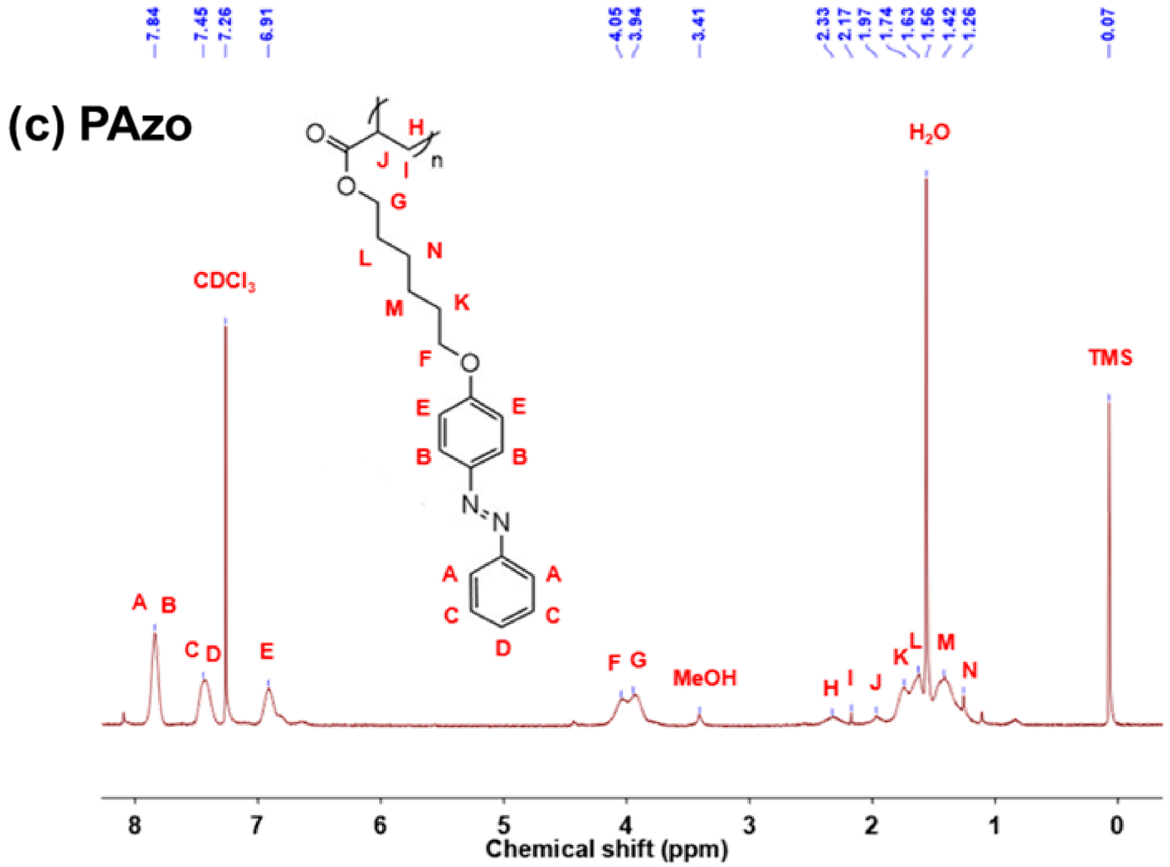

Figure S2. Synthetic routes of (a) azobenzene-containing polymer (PAzo) and ^{1}H nuclear magnetic resonance (^{1}H NMR) spectra of (b) Azo monomer (Azo3) and (c) PAzo.

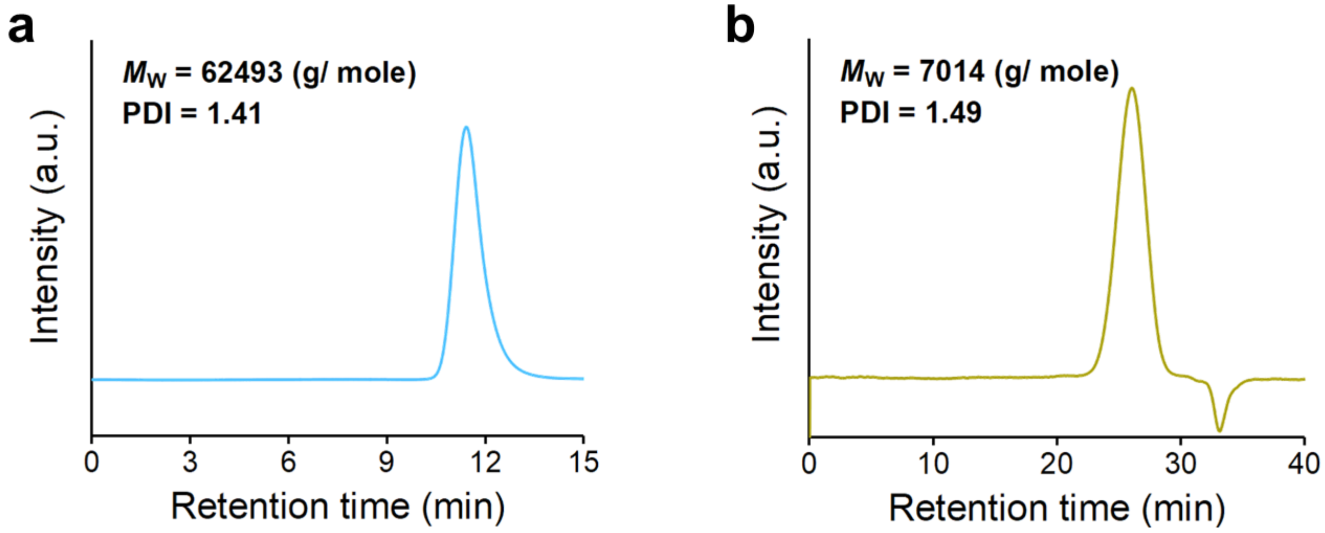

Figure S3. GPC data of (a) TPEE, calibrated with PMMA-standards, with trifluoroethanol (TFE) as the mobile phase and (b) PAzo, calibrated with PS-standards, with tetrahydrofuran (THF) as the mobile phase.

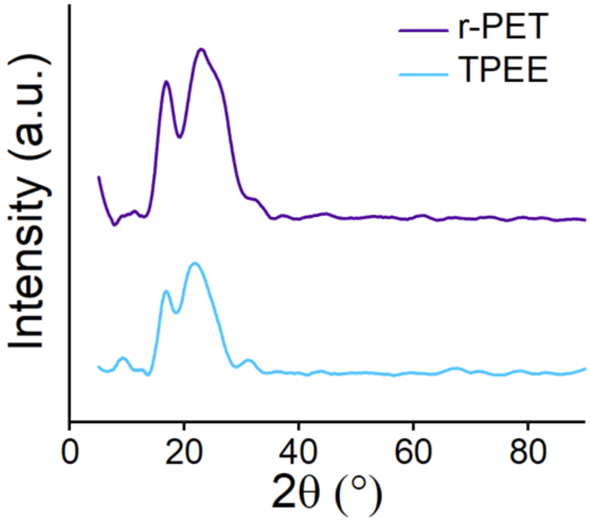

Figure S4. WAXRD spectra of r-PET and TPEE films.

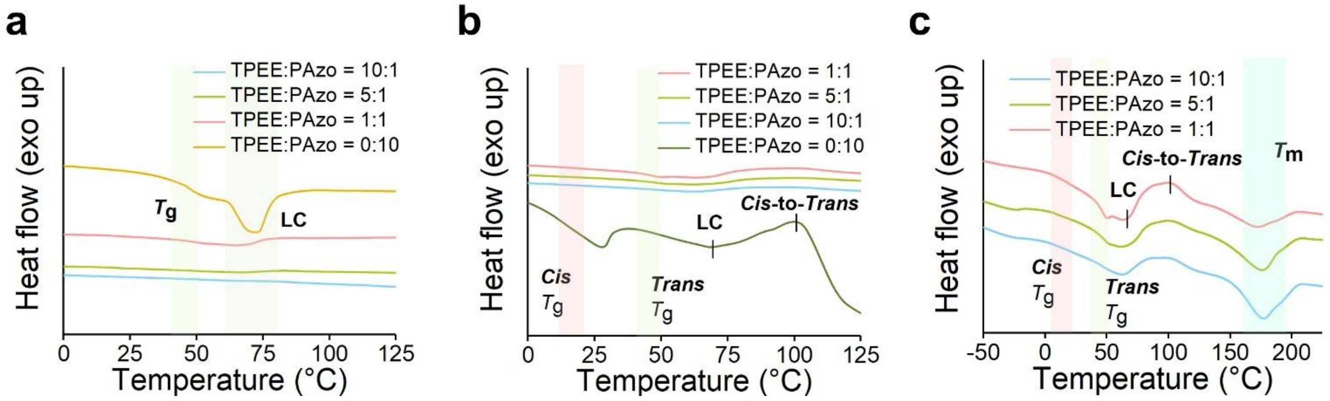

Figure S5. DSC curves of TPEE/PAzo blends with different blend ratios: (a) before UV irradiation, (b) after UV irradiation, and (c) TPEE/PAzo blend fibers after UV exposure.

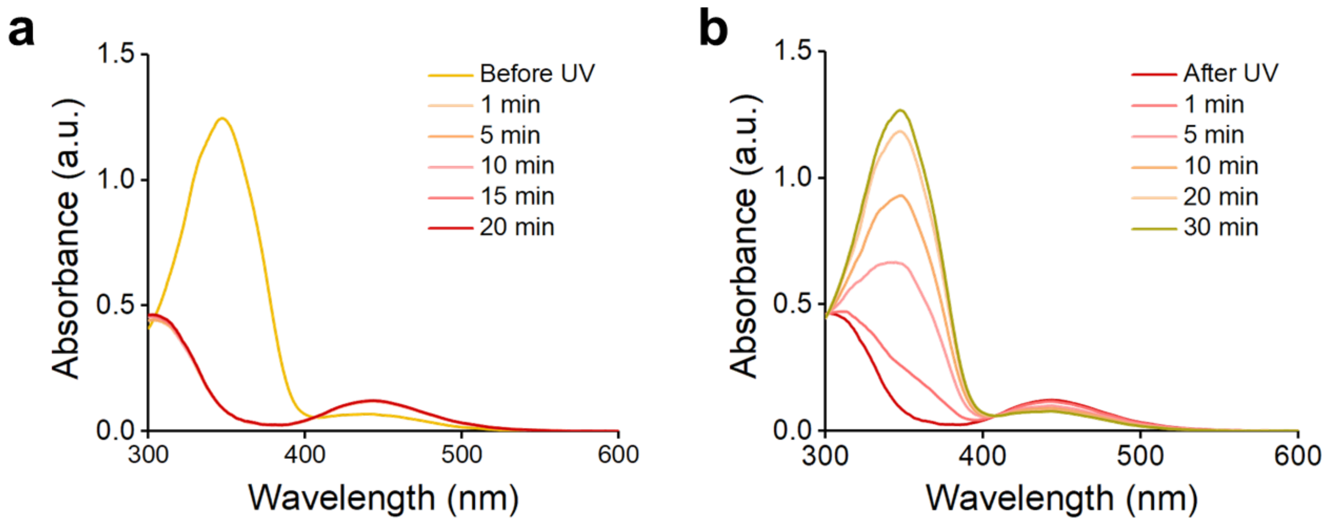

Figure S6. UV–Vis absorption spectra of (a) PAzo solution under UV and (b) visible light exposure over varying time intervals.

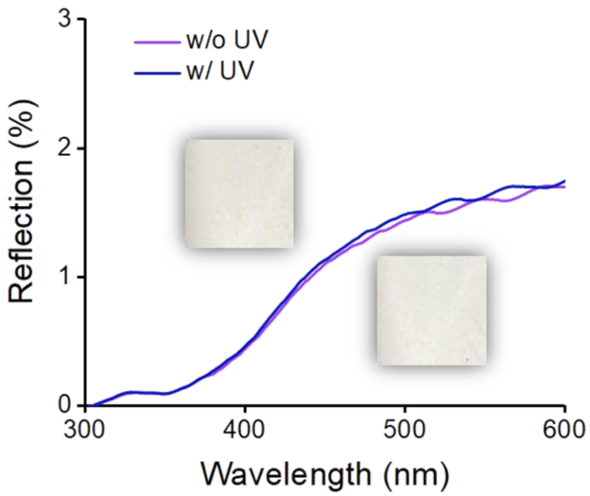

Figure S7. UV–Vis reflection spectra of pure TPEE fibers without and with UV irradiations.

Table S1. Maximum stress (MPa) values of pristine and aged (180 days) TPEE/PAzo blend fibers before and after UV irradiations

**TPEE:PAzo blend ratio**	**Pristine (MPa)**		**After 180 days (MPa)**	
	Trans	***Cis***	***Trans***	***Cis***
10:1	7.88 ± 0.81	7.99 ± 1.55	4.23 ± 0.52	4.77 ± 0.16
5:1	7.62 ± 0.41	8.10 ± 0.45	5.09 ± 0.47	5.79 ± 0.84
1:1	5.21 ± 0.20	5.54 ± 0.22	3.39 ± 0.56	3.67 ± 0.31

Table S2. Maximum strain (%) values of pristine and aged (180 days) TPEE/PAzo blend fibers before and after UV irradiations

TPEE:PAzo blend ratio	Pristine (%)		After 180 days (%)	
	Trans	***Cis***	***Trans***	***Cis***
10:1	198.94 ± 25.26	240.49 ± 10.91	156.34 ± 5.82	193.26 ± 4.31
5:1	169.41 ± 24.84	218.62 ± 21.11	126.45 ± 7.46	154.86 ± 7.19
1:1	108.09 ± 10.09	131.68 ± 10.52	76.33 ± 9.79	84.17 ± 3.13

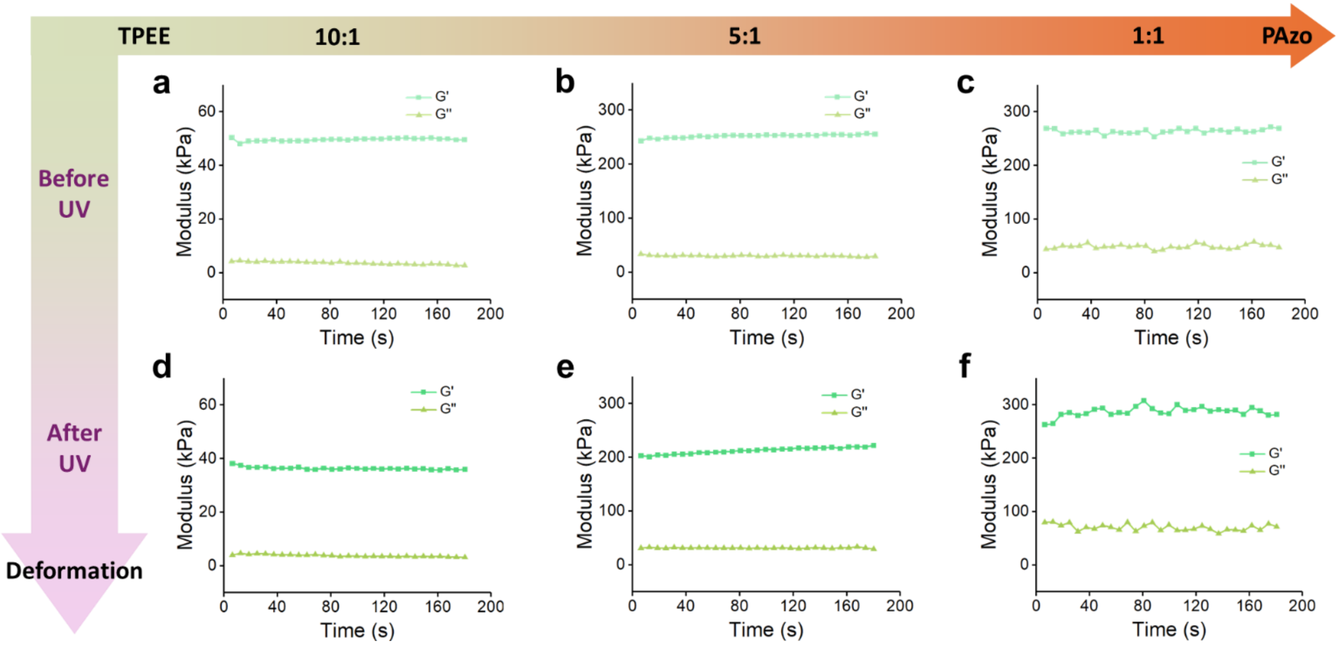

Figure S8. Rheological analysis of the TPEE/PAzo blend fibers showing the storage modulus (G′) and loss modulus (G″) over time at an angular frequency of $\omega = 10$ rad/s and 25 °C, with compression force fixed at 5 N. Blend ratios of TPEE:PAzo are (a, d) 10:1; (b, e) 5:1; and (c, f) 1:1, measured before and after 20 min of UV irradiations.

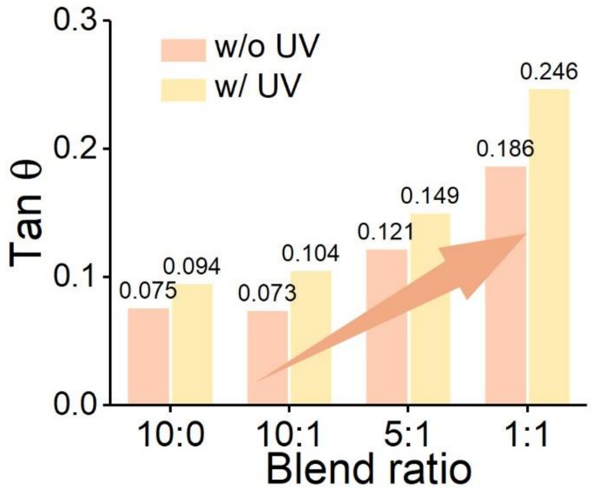

Figure S9. Comparison of tan(θ) values for TPEE/PAzo blend fibers with varying TPEE:PAzo ratios.

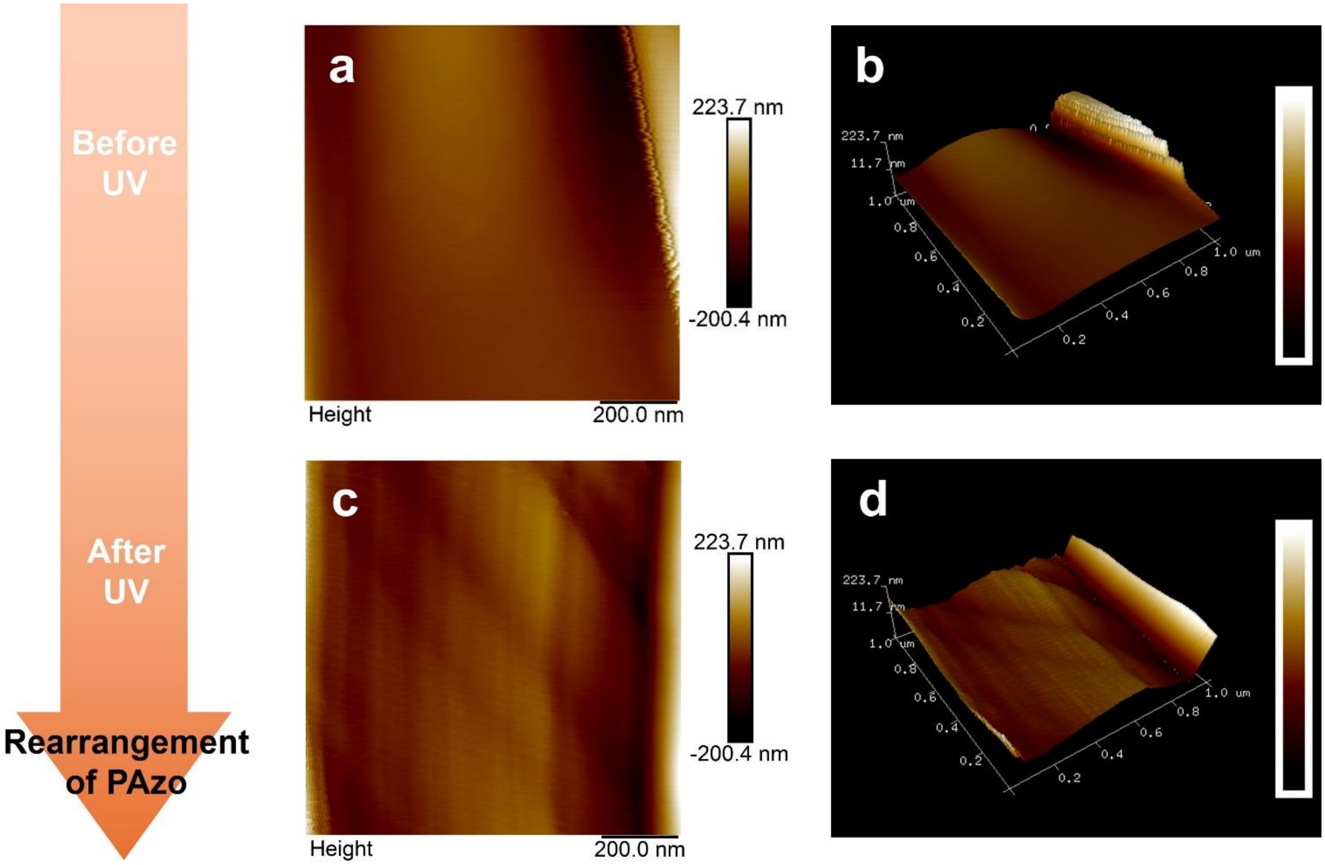

Figure S10. (a–d) Atomic force microscopy (AFM) images of a single TPEE/PAzo blend fiber with a ratio of 5:1, captured in tapping mode. 2D (a, c) and 3D (b, d) surface topographies are shown before (a, b) and after (c, d) 20 minutes of UV irradiation.

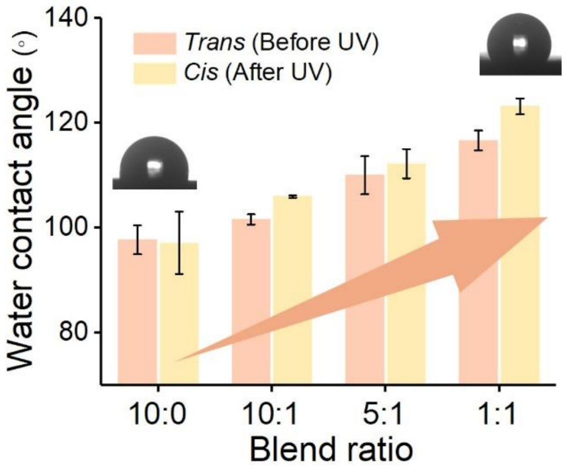

Figure S11. Water contact angle (WCA) measurements of TPEE/PAzo blend fibers with varying blend ratios (10:0, 10:1, 5:1, and 1:1), comparing values before and after UV irradiation.

Table S3. Healing efficiency (%) of TPEE/PAzo blend fibers with different blend ratios after photo-induced lap shear healing. The healing efficiency is calculated by dividing the maximum strain of the healed sample by that of the undamaged fiber

**TPEE:PAzo blend ratio**	**Healing efficiency (%)**	
	**Unaged fibers**	**Aged fibers**
10:1	11.8 ± 1.1	14.3 ± 1.3
5:1	50.7 ± 1.3	54.3 ± 2.8
1:1	20.5 ± 4.5	26.2 ± 7.4

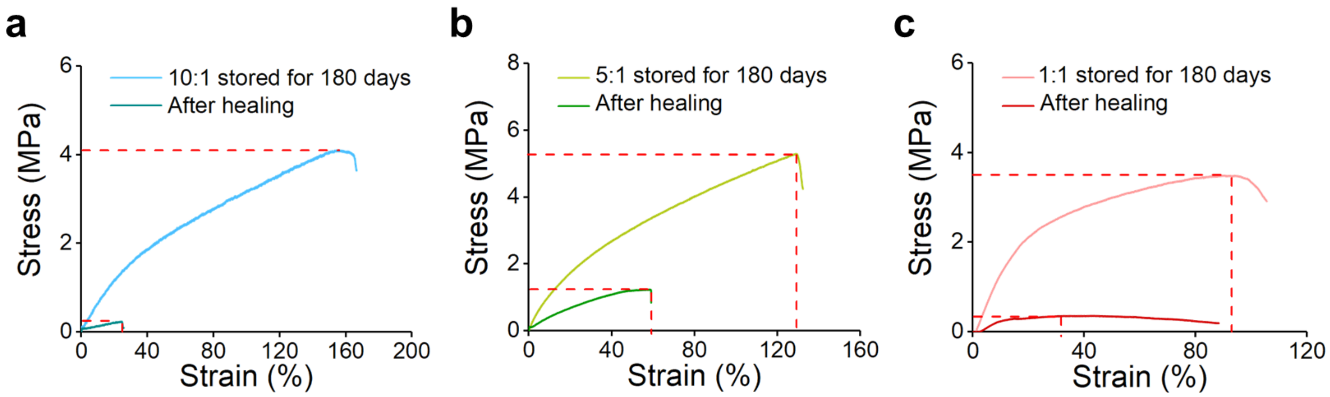

Figure S12. Tensile stress–strain curves of aged fibers (stored for 180 days), comparing the original (undamaged) and healed samples at different TPEE:PAzo blend ratios: (a) 10:1, (b) 5:1, and (c) 1:1.

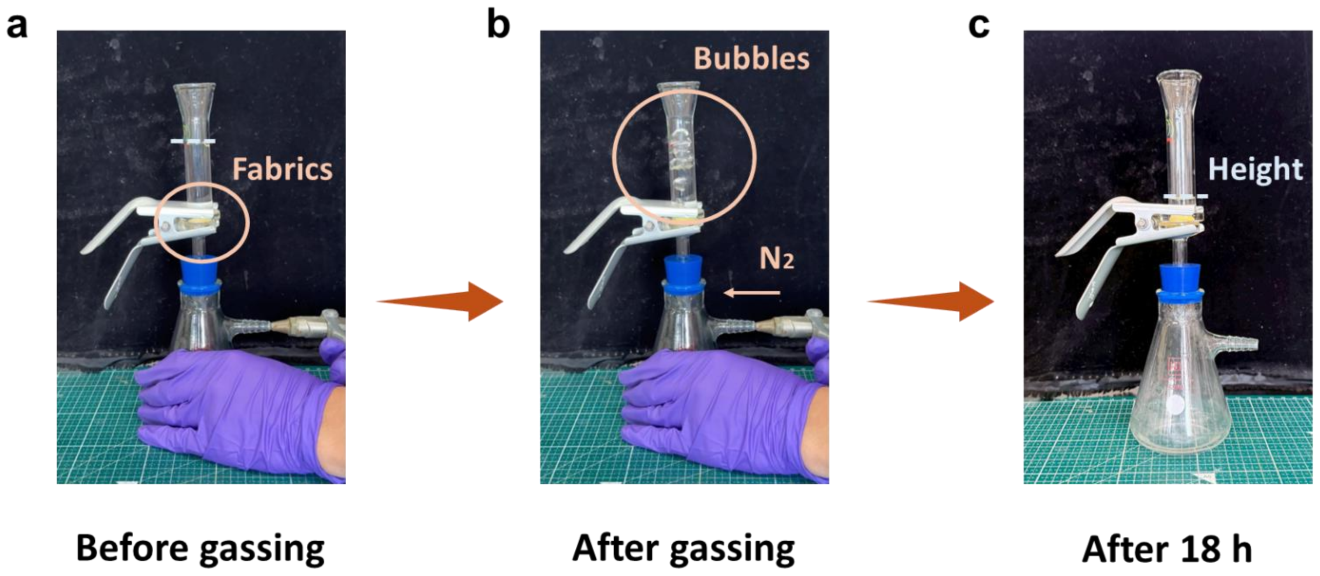

Figure S13. Breathability and water resistance test for TPEE/PAzo blend fabrics. In the test, the fabrics are placed over the neck of a filtering flask and water is poured on top. (a) Before introducing high-pressure N_2 gas into the flask; (b) after introducing high-pressure N_2 gas through the flask port; (c) condition of the setup after 18 h.

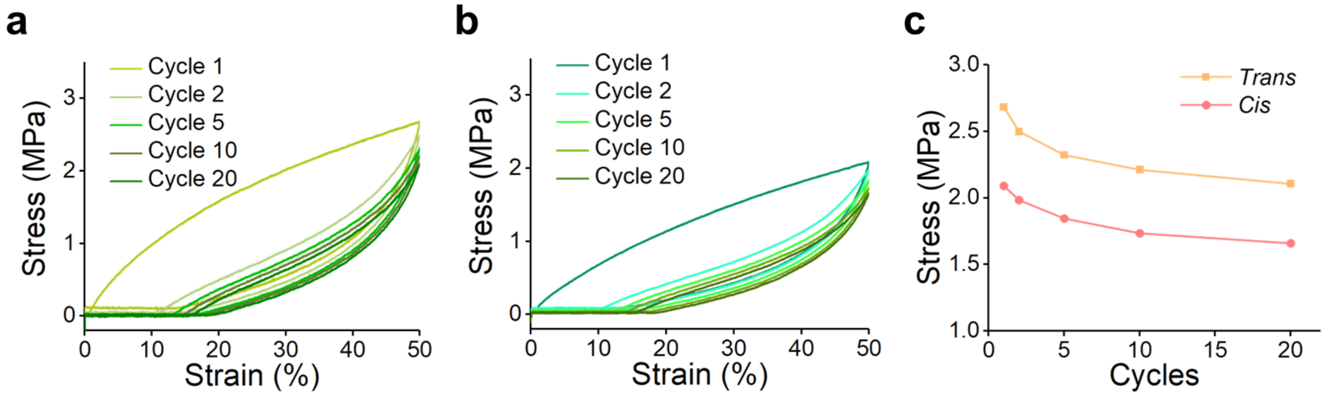

Figure S14. Cyclic tensile test of TPEE/PAzo blend fibers with a blend ratio of 5:1. (a) *Trans* state; (b) *cis* state; (c) comparison of stress values at 50% strain in both *trans* and *cis* states after 20 cycles.

Reference

(1) Y.-F. Chen, M.-R. Huang, Y.-S. Hsu, M.-H. Chang, T.-Y. Lo, B. Gautam, H.-H. Hsu, J.-T. Chen. Photo-Healable Fabrics: Achieving Structural Control via Photochemical Solid–Liquid Transitions of Polystyrene/Azobenzene-Containing Polymer Blends. *ACS Appl. Mater. Interfaces.* **2024**, *16*, 29153.
